# Best Practices in the Management of Infectious Complications for Patients With Cancer

**Published:** 2017-04-01

**Authors:** James S. Lewis II

**Affiliations:** Oregon Health & Science University, Portland, Oregon

## Abstract

As antibiotic resistant bacteria are becoming ubiquitous, managing infection becomes more complex. Optimizing preventive measures, early diagnosis, and management of infection is of the highest priority in cancer care.

Biosimilars of filgrastim for the treatment of febrile or prolonged neutropenia have already arrived on the market and will continue to increase in use based on price advantage. According to James S. Lewis II, PharmD, FIDSA, of Oregon Health & Science University, Portland, this is good news for patients and health-care systems, but it’s just one step in a complicated treatment plan. Bacterial pathogens also represent a serious threat to patients with cancer.

"Drug-resistant bugs are increasingly problematic," said Dr. Lewis. "Appropriate prophylaxis and appropriate vaccination save lives in profoundly immunocompromised patients or those who are going to be immunocompromised for a long time."

At the 2016 JADPRO Live conference, Dr. Lewis discussed the role of myeloid growth factors in the prevention and treatment of febrile neutropenia and the impact of worrisome developments with respect to superinfection and drug resistance. He also described treatments for infections commonly seen in patients with cancer and what advanced practitioners should be aware of when strategizing a treatment approach.

## BIOSIMILAR VS. GENERIC GROWTH FACTORS: WHAT’S THE DIFFERENCE?

Although generics are small, simple molecules that are easy to manufacture, biosimilars are extremely large and complicated proteins that are produced from living systems and are therefore challenging to replicate (US Food and Drug Administration [FDA], 2016). "These are massive proteins, so the complexity of the molecule is much different than anything we see with standard generics," said Dr. Lewis.

Another major difference between biosimilars and generics is interchangeability: None of the four approved biosimilars (filgrastim-sndz [Zarxio], infliximab-dyyb [Inflectra], etanercept-szzs [Erelzi], and Amjevita [adalimumab-atto]) is currently considered interchangeable with reference products. Because of the size and complexity of these molecules, the guidelines of the NCCN Clinical Practice Guidelines In Oncology (NCCN Guidelines®) recommend that patients remain on the same product throughout treatment (NCCN, 2016a).

"Let’s be real," said Dr. Lewis. "If you actually switch up a dose of biosimilar filgrastim from original filgrastim, you’re not likely to hurt the patient, but whatever agent starts a patient’s regimen should ideally complete the remainder of therapy."

"For other intents and purposes, though, you should think of biosimilars as complicated generics," he added. "Given the FDA’s stringent standards for biosimilar approval and the potential for significant cost savings, clinicians should encourage rapid integration of these products."

Filgrastim-sndz has been approved for the same five indications as filgrastim: (1) patients with cancer who are receiving myelosuppressive chemotherapy; (2) patients with acute myeloid leukemia (AML) receiving induction or consolidation chemotherapy; (3) patients with cancer undergoing bone marrow transplantation; (4) patients with cancer undergoing autologous peripheral blood progenitor cell collection and therapy; and (5) those with severe chronic neutropenia. Tbo-filgrastim (Granix) has only been studied for prophylaxis and has been approved for the reduction in duration of severe neutropenia in nonmyeloid malignancies.

## WHO NEEDS MYELOID GROWTH FACTORS?

According to Dr. Lewis, the intensity of the chemotherapy regimen drives febrile neutropenia rates. "The harder you’re going to hit these patients and the more marrow-toxic these agents are, the deeper the neutropenia is likely to become," he explained. "Myeloid growth factors are recommended as prophylaxis for patients receiving any chemotherapy regimen with greater than a 20% risk of febrile neutropenia."

Other risk factors for febrile neutropenia include age (especially patients older than 65 who receive full-dose chemotherapy); previous chemotherapy or radiotherapy; preexisting neutropenia or tumor involvement of bone marrow; poor performance status; and a high number of comorbidities (NCCN, 2016a).

"The frailer the patient becomes, the more you need to think about using these agents," said Dr. Lewis. "When these agents are used in appropriate patients, they reduce infection rates, hospital length of stay, and the duration and severity of neutropenia. There’s a lot of upside."

Although the decision is trickier for intermediate-risk patients, he admitted, "if it’s my mom or dad, I’d use these agents." For patients presenting with infections, however, it’s more difficult to find good data, but myeloid growth factors seem to be associated with a shorter length of stay and a shorter duration of neutropenia.

"There is some suggestion of decrease in infection-related mortality, which is all you can ask these compounds to do," stated Dr. Lewis. "If a patient has already received pegfilgrastim (Neulasta), though, therapeutic use of myeloid growth factors is not going to be a benefit."

## ANTIBIOTIC-RESISTANT BACTERIA

As Dr. Lewis reported, growth factors have added significance because of the emergence of antibiotic-resistant bacteria. Nationally and internationally, he said, a variety of bacterial pathogens are becoming increasingly difficult to treat.

"I’m becoming a huge growth factor fan," said Dr. Lewis, "because they can prevent infections, which lead to antibiotics, which lead to drug-resistant bacteria."

The following bacterial pathogens represent urgent threats: *Clostridium difficile*; carbapenem-resistant Enterobacteriaceae (CRE); and drug-resistant *Neisseria gonorrhoeae*. Those pathogens representing serious threats include: multidrug-resistant *Pseudomonas aeruginosa* and Acinetobacter spp; extended-spectrum beta-lactamase (ESBL)-producing Enterobacteriaceae; methicillin-resistant *Staphylococcus aureus* (MRSA) and vancomycin-resistant enterococcus (VRE); and various drug-resistant species (Campylobacter spp, *Staphylococcus pneumoniae*, Salmonella spp, *Mycobacterium tuberculosis*, Shigella spp). Pathogens associated with health-care–associated infections are listed in the Table.

**Table:**
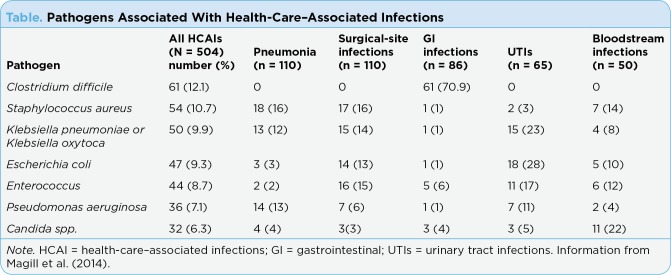
Pathogens Associated With Health-Care–Associated Infections

"In New York City, up to 40% of Klebsiella isolates are now carbapenem-resistant," said Dr. Lewis. "Carbopenems are the Godzillas of the antibacterial world. When you’re resistant to them, you’re resistant to all beta-lactams. It doesn’t leave you with much." As Dr. Lewis reported, patients found to have infections with carbapenem-resistant bacteria have a mortality rate approaching 40%, and they’re far from the only threat.

Resistant organisms cause more than 2 million illnesses and at least 23,000 deaths each year in the United States (Centers for Disease Control and Prevention [CDC], 2013). The good news, however, is that the problem is in part preventable.

According to the CDC, up to 70% fewer patients will experience CRE in 5 years if facilities coordinate to protect patients (CDC, 2015). "Preventing infections and improving antibiotic prescribing could save 37,000 lives from drug-resistant infections over 5 years," said Dr. Lewis. "It will also help reduce hospital length of stay and health-care costs."

"We have to be careful not only with how we use antibiotics in our patients, but also how we use them as a society. We are down to the last few options," he acknowledged.

## WHO IS AT RISK?

Patients at risk for colonization and subsequent infections with multidrug-resistant gram-negative organisms are those with the following characteristics: previous exposure to broad-spectrum antibiotics; exposure to an increasing number of antibiotics; increasing age; increasing chronic disease score; previous stay in the intensive care unit; chronic obstructive pulmonary disease; and increasing duration of hospitalization (Harris et al., 2007; Papadimitriou-Olivgeris et al., 2012).

"We need to be doing everything we can to minimize antibiotic exposures in these patients and to ensure that we get their white cells back in a timely fashion to reduce their risk of infection," said Dr. Lewis.

Nevertheless, time is critical with respect to antibiotic initiation, as well, he added. "When a patient appears septic in your emergency room or outpatient infusion center, time is of the essence," he said. "For a patient with sepsis, every hour of delay in effective antibiotic therapy mortality risk increases by 7% (Kumar et al., 2006). You need to choose appropriate antibiotics and get them on board in a timely fashion."

## NEUTROPENIA DRIVES INFECTIONS

"The immune system is your friend," said Dr. Lewis, and when it’s compromised, a patient is more likely to develop infection. In addition, long-term data have shown that neutropenia drives infections; the longer neutropenia persists, the higher the risk (Carlisle, Gucalp, & Wiernik, 1993). Mucositis and impaired organ function due to tumor burden are also factors that predispose patients to risk. However, what providers don’t consider frequently enough, he said, are medications.

"The problem with all the new medications is that we don’t know the full extent of the side-effect profile until they’re out in clinical practice for a while," said Dr. Lewis, who added that corticosteroids can result in profound immunosuppression, as well. "We get so comfortable flinging big doses of steroids around, sometimes we forget exactly how immunosuppressant these agents can be."

With respect to neutropenia, Dr. Lewis emphasized the speed of neutrophil decline. "The speed with which a patient’s white blood cell count is dropping tends to predict the duration and severity of neutropenia," he explained. "When these numbers decline quickly, it’s time to see if you can do anything about it, and you should have a very low threshold to start thinking about infections."

Guidelines from the National Comprehensive Cancer Network® (NCCN®) for initial risk assessment for patients with febrile neutropenia are shown in the Figure.

**Figure:**
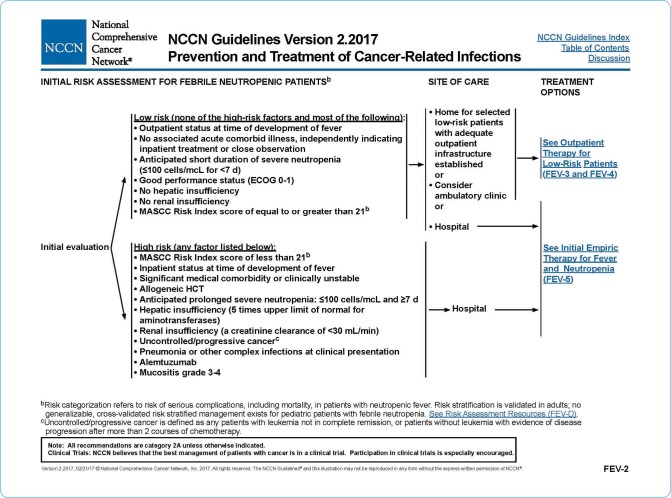
Reproduced with permission from the NCCN Clinical Practice Guidelines in Oncology (NCCN Guidelines®) for Prevention and Treatment of Cancer-Related Infections. Version 2.2017 © 2017 National Comprehensive Cancer Network, Inc. All rights reserved. The NCCN Guidelines® and illustrations herein may not be reproduced in any form for any purpose without the express written permission of the NCCN. To view the most recent and complete version of the NCCN Guidelines, go online to NCCN.org. NCCN makes no warranties of any kind whatsoever regarding their content, use or application and disclaims any responsability for their application or use in any way.

## PREVENTING INFECTIONS IN IMMUNOCOMPROMISED PATIENTS

According to the NCCN Guidelines®, quinolones are the best studied agents with respect to prophylaxis during neutropenia (NCCN, 2016b). Data have shown that quinolones lead to fewer gram-negative infections, said Dr. Lewis, but prophylaxis is not recommended for patients with a low risk of infection. Instead, quinolone prophylaxis should be reserved for intermediate- and high-risk patients, patients expected to have neutropenia lasting longer than 7 days, and patients who have had problems with prior chemotherapy leading to febrile neutropenia. When dealing with stem cell transplant patients, Dr. Lewis urged providers to remember pneumococcus. "*Streptococcus pneumoniae* remains as nasty as ever, and penicillin prophylaxis is encouraged out to a year after all allogeneic stem cell transplants," he indicated. "Make sure it’s continued until immunosuppression for graft-vs.-host disease is complete."

## VACCINES

With respect to vaccines, Dr. Lewis offered the following general rule: "If it moves, vaccinate it!" In other words, good vaccines are available, so providers should make better use of them and follow the NCCN Guidelines (NCCN, 2016b).

"This is an area that we need to pay a lot of attention to," he encouraged. "Vaccinate patients who you know are going to be receiving chemotherapy regimens ahead of time. Even if you only get a partial response, it’s far better than no response, which is what you’ll get if you do not vaccinate."

However, some of these vaccines, such as those against Varicella zoster, are live vaccines, he cautioned. "You don’t want to give live vaccines to people who are profoundly immunocompromised," he added. "Make sure you refer to that guideline document."

Finally, Dr. Lewis recommended that providers stay informed of their institution’s antibiogram. "Find out what grows in your hospital and what your resistance rates are. It’s important to know what’s going on at your institution," he concluded.
